# Editorial: The immunological role of platelet activation in the pathophysiology of COVID-19

**DOI:** 10.3389/fimmu.2023.1285355

**Published:** 2023-09-25

**Authors:** Theresa Marie Rossouw, Etheresia Pretorius, Geoffrey D. Wool

**Affiliations:** ^1^ Department of Immunology, University of Pretoria, Pretoria, South Africa; ^2^ Department of Physiological Sciences, Stellenbosch University, Stellenbosch, South Africa; ^3^ Department of Biochemistry and Systems Biology, Institute of Systems, Molecular and Integrative Biology, Liverpool, United Kingdom; ^4^ Department of Pathology, The University of Chicago, Chicago, IL, United States

**Keywords:** platelets, SARS-CoV-2, inflammation, immuno-thrombosis, vaccine-induced immune thrombocytopenia and thrombosis

Platelets are increasingly recognized for their role as mediators of immune response and inflammation. As major components of the hematological system, they form an important bridge between immunity and coagulation. When platelets become hyperactivated in response to an infection, patients can develop immuno-thrombosis and coagulopathy. These derangements of hemostasis are particularly relevant in the context of infection with the novel coronavirus, SARS-CoV-2, and the subsequent development of coronavirus disease, COVID-19, a disease in which thromboembolic events are an important cause of morbidity and mortality.

This Research Topic focuses on the immunological role of platelet activation in the pathophysiology of COVID-19 as well as Vaccine-induced Immune Thrombocytopenia and Thrombosis (VITT) in *in vitro* and *in vivo* environments.

Jevtic and Nazy provide an elegant review of platelet hyperactivation in COVID-19. They describe how the disease is associated with changes in platelet surface markers, secretion responses, and gene expression as well as increased circulating platelet-leukocyte aggregates. Immune complexes appear to contribute significantly to this platelet-activating effect; the antigen specificity and additional characteristics of these immune complexes remain to be determined.

Platelet activation *in vivo* in COVID-19 patients can lead to reduced responsiveness to additional *in vitro* stimuli, due to functional exhaustion of these anucleate corpuscles. Apostolidis et al. show that COVID-19 is associated with increased markers of basal platelet activation and reduced agonist responsiveness. Exposure to COVID-19 plasma increases donor platelet activation and, in a flow model, causes more platelet adhesion. The ability of patient plasma to activate donor platelets correlates with worsened patient outcomes. These effects occur through FcgRIIa-Syk and C5a-C5aR pathways; this identifies Syk kinase inhibitors and C5 inhibitors as therapeutics with potential promise for COVID-19 thromboinflammation.

The role of complement activation is also explored by Perico et al. They describe a novel mechanism through which SARS-CoV-2-derived S1 protein sufficiently induces inflammatory and thrombogenic processes in the microvasculature. They demonstrate that the *in vitro* activation of endothelial cells with S1 protein, *via* ACE2, lead to leukocyte recruitment and C3 and C5b-9 deposition on endothelial cells, along with C3a and C5a generation that further magnify spike-induced complement activation. They propose that this process possibly recapitulates the systemic thromboembolic complications observed in severe COVID-19 cases, hence strengthening the argument for the use of complement inhibitors.

Capozzi et al. investigate patients with COVID-19 who experienced ischemic stroke. Immunoglobulin from such patients, particularly when containing antiphospholipid antibodies, is shown to activate donor platelets through phosphorylation of ERK and p38 as well as cause aggregation and ATP secretion.

Extracellular vesicles (EV) in biological fluids can be identified through light scatter properties and the presence of phosphatidylserine (PS) on their outer membrane leaflet. Annexin A5 binding is often used to identify PS positive particles. Jacob et al. report a significant increase in annexin A5 avid circulating EV (total plasma as well as endothelial- and platelet- derived EV) in COVID-19 ICU patients. Such microparticles are associated with increased thrombotic events.

Overall, these studies demonstrate the significant interplay of the humoral immune system and platelet activation in COVID-19, contributing to the high rate of thromboembolism seen in this disease.

The blood of people living with HIV (PLWH) are known to be hypercoagulable and they might therefore be particularly vulnerable to the thromboembolic complications of SARS-CoV-2. In an insightful study, van der Mescht et al., assess platelet and endothelium-associated cytokines, chemokines, and growth factors in 174 patients hospitalised with COVID-19. HIV co-infection was not associated with increased intensity of the inflammatory response. They do, however, report an interesting association between increased levels of the angiogenic factor, plasma vascular endothelial growth factor (VEGF), with SARS-CoV-2/HIV co-infection. Of note, PLWH with low CD4 counts or uncontrolled HIV replication, had lower levels of VEGF concentrations, possibly indicating an inappropriate immune response.

Rossouw et al. review the interaction of platelets with the vascular endothelium in the context of COVID-19-related myocardial injury. They describe the interaction of the viral spike protein with endothelial ACE2 together with alternative mechanisms that involve nucleocapsid and viroporin in the generation of a generalized endotheliitis. They further explain how platelet-derived calcium-binding proteins, SA100A8 and SA100A9, intensify endothelial activation and dysfunction. These events create a SARS-CoV-2–driven cycle of intravascular inflammation and coagulation, which contributes significantly to a poor clinical outcome in patients with severe disease.

Finally, three articles explore the immunological role of platelet activation in the pathophysiology VITT.

In an unprecedented head-to-head comparison between the Oxford/AstraZeneca [ChAdOx1] (AZ) and mRNA vaccines, Ostrowski et al. present original data about the diverse responses elicited by different vaccines. Their findings reveal enhanced inflammation, platelet activation, and thrombin generation following AZ vaccination and shed light on potential triggers and mechanisms underlying complications like VITT.

Pang et al. in a hypothesis and theory paper, venture into the uncharted territory of anionic substances binding to platelet factor 4 (PF4) in the context of VITT induced by the ChAdOx1-S vaccine. They identify five potential candidates within the vaccine that could trigger VITT, with negatively charged impurity proteins emerging as the most likely instigator. By unravelling the molecular interactions between these anionic substances and PF4, the authors shed light on the intricate web of events that give rise to VITT.

These hypotheses lay the groundwork for a deeper understanding of VITT’s, offering a potential avenue for risk assessment and intervention strategies to ensure the safety of vaccine recipients.

In a succinct mini review, Hirsch et al. explore the interplay between platelets and neutrophils, both in the context of COVID-19 and VITT. The review underscores the central roles played by hyperactivated platelets and neutrophil extracellular traps in the coagulopathy associated with COVID-19. The authors highlight the emergence of a new paradigm in thrombophilia, catalysed by auto-antibody formation in response to adenoviral vector vaccines.

The collective insights from the papers, spanning original data to theoretical exploration and concise synthesis, featured in this Frontiers of Immunology Research Topic, underscore the need for multidisciplinary research and edge us forward towards unravelling the complexities of platelet activation in the context of infection.

## Author contributions

TR: Writing – original draft, Writing – review & editing. EP: Writing – original draft, Writing – review & editing. GW: Writing – original draft, Writing – review & editing.

